# Self-exclusion and breaching of self-exclusion from gambling: a repeated survey study on the development of a nationwide self-exclusion service

**DOI:** 10.1186/s12954-023-00822-w

**Published:** 2023-08-08

**Authors:** A. Håkansson, N. Komzia

**Affiliations:** Department of Clinical Sciences Lund, Psychiatry, Lund University, Lund, Sweden

**Keywords:** Gambling disorder, Problem gambling, Self-exclusion, Harm reduction, Behavioral addiction

## Abstract

**Background:**

Voluntary self-exclusion from gambling is a common harm reduction tool in individuals with a gambling disorder. Previous data have demonstrated that many gamblers breach their own self-exclusion, typically through other online services outside the jurisdiction in which they are self-excluded. The present study aimed to carry out a new follow-up measure—similar to previous studies in the same setting—of self-exclusion and its breaching in Sweden, in order to allow for the follow-up assessment of a nationwide, multi-operator self-exclusion system introduced in Sweden in 2019.

**Methods:**

A web survey to the web panel of a market survey company addressed 1505 past-year gamblers, who responded to a number of questions about gambling habits, including screening for gambling problems using the Problem Gambling Severity Index and self-exclusion-related items corresponding to previous studies.

**Results:**

Nine percent of past-year gamblers had self-excluded using the *Spelpaus* service. In logistic regression, self-exclusion was significantly associated with gambling problems, past-year online casino gambling, and absence of online poker gambling. Among self-excluders, 49 percent had ever gambled despite being self-excluded. Among those breaching their self-exclusion, the most common gambling types during self-exclusion were online casino (82 percent), sports betting (47 percent) and lotteries (43 percent).

**Discussion:**

Self-exclusion remains a popular harm reduction tool against problem gambling, more common than in previous studies, mostly in individuals with recent gambling problems and in online casino gamblers. However, breaching self-exclusion is somewhat more common than in previous research. Online casino represents the most common means of self-exclusion breaching. Policy-making in the area needs to further address the risk of breaching one’s self-exclusion and may further address the risk of overseas gambling.

## Background

Gambling disorder (GD [1]) is a behavioral addictive disorder representing problematic gambling for money in a pattern often characterized by increased tolerance and loss of control, an urge to gamble in order to compensate for recent gambling losses (‘chasing losses’ behavior), financial dependence on others, and severe consequences for the psycho-social well-being of oneself and one’s concerned significant others. Consequences can often be devastating for affected individuals and families [2, 3]. In the management of GD, voluntary self-exclusion is a frequent and well-established self-help measure, aiming to reduce the harm associated with gambling, and to obtain discontinuation of gambling in individuals who perceive difficulties to control their own gambling practices [4–6]. In recent years, such self-exclusion services in some settings have increasingly involved several gambling modalities or several gambling operators instead of only one. That is to help gamblers prevent themselves from breaching their self-exclusion through other gambling operators outside of the venue or website from which they have self-excluded [7].

The Swedish self-exclusion service allows for any person to self-exclude from all licensed gambling operators simultaneously, including both land-based and online gambling providers. *Spelpaus* is a nationwide service, operated by a government authority and independent of gambling operators. The system allows the person choosing to self-exclude to do so through a safe, state-operated platform managed by a government authority (The Swedish Gambling Authority), instead of visiting a gambling operator’s website. The *Spelpaus* service offers the possibility to self-exclude either for one month, three months, six months, or 12 months, and each period can voluntarily be followed by a next one. Every log-in attempt on a licensed Swedish gambling operator is electronically run against this self-exclusion register, such that a self-excluded individual is unable to log onto the gambling sites of all licensed operators and prevented from entry at the three state-owned land-based casinos in Sweden. Thus, this includes sports betting in online or land-based gambling venues, online casino and bingo games, online card games, online scratch tickets, and land-based electronic gambling machines. Gambling types not included in the system include the buying of lottery tickets in grocery stores and similar, the entry into land-based bingo venues, and gambling on a type of limited-stake casino table games offered in some restaurants (so-called restaurant casinos), gambling types which, however, are very rarely represented in treatment-seeking patients with a GD in this setting [8].

This system was introduced along with a new gambling market in 2019, and the *Spelpaus* service has been described previously. In a highly online-based gambling market, the need for a new legislation included the need for a transfer from a largely overseas-based gambling market outside of judiciary control in Sweden, to a system requiring preventive measures to be taken by gambling operators operating in this market. As part of this, the new legislation allowed a number of licensed gambling operators, provided they respect a number of preventive legal measures including the mandatory adherence to the *Spelpaus* system [9, 10]. To the best of our knowledge, fully effective self-exclusion programs are not available, and online gambling, making overseas gambling markets possible for anyone to reach, may further present a major challenge to self-exclusion as a harm-reducing strategy.

In a Swedish gambling disorder treatment unit (the regional GD treatment unit of Skåne county within the addiction psychiatry department of the health care services, as described in previous literature [8]), a majority of treatment-seeking patients in 2021 had self-excluded from gambling, and the majority among them had continued to gamble during their self-exclusion period. Gambling on foreign online operators was by far the most common way of breaching self-exclusion [11]. Likewise, in a previous web survey addressing past-year gamblers, 38 percent of those who had ever self-excluded (since the introduction of the present self-exclusion service in 2019) had breached their self-exclusion and gambled despite it, most commonly on online casinos [9].

Thus, the overall rationale behind the present study is the concerning rates of gambling relapse despite self-exclusion, and although low absolute numbers hitherto have made such conclusions difficult, data have reported a possible association between the breaching of one’s self-exclusion and higher rates of treatment needs and psychological distress [9], and breaching typically occurs in the gambling types most commonly associated with treatment-requiring GD [8]. Data describing the function and possible limitations of a nationwide self-exclusion service is needed, in order to inform policy makers and other stakeholders in the area in order to optimize policy and preventive measures. More than three years into a new gambling market regulation, including the nationwide self-exclusion service, there is reason to provide a follow-up to previous figures describing the prevalence and breaching of voluntary self-exclusion in Swedish gamblers. There are also, to the best of our knowledge, no studies from other settings which describe—over time—the outcomes of a self-exclusion program that is multi-operator, state-owned and involves nationwide land-based and online operators, and thereby both covers a very large number of potential gambling opportunities but which may also still be limited by the risk of breaching through illegal or overseas gambling. Thereby, the present study topic is original and may be of relevance to disseminate to other countries considering a potentially similar model. Given the literature highlighting the potential limitations of self-exclusion systems in general [7], there is need to evaluate this service.

Therefore, the present study used a study design similar to the 2020 and 2021 survey studies assessing self-exclusion and other gambling practices [9], and aimed to study the rates of self-exclusion, breaching of self-exclusion, and correlates of self-exclusion and its breaching in past-year gamblers in Sweden. Specific research questions of the study are the following: (1) How common is self-exclusion through a nationwide, multi-operator self-exclusion service among gamblers in Sweden and which factors are associated with the choice to self-exclude; (2) How common is breaching of self-exclusion, i.e. gambling on other types of gambling modalities during one’s self-exclusion period, and which factors are associated with such breaching; and (3) How do these findings over time compare to earlier observations from the present setting and using a similar methodology?

## Methods

A web survey was carried out in Sweden in May, 2022, addressing individuals enrolled with the web panel of a market survey company (Ipsos). The survey addressed individuals with any occasion of gambling for money during the past 12 months. Recruitment aimed to reach a gender and age distribution comparable to that of the general population, and to continue until 1500 individuals were included. When recruitment stopped, a total of 1505 unique participants had been included. In comparison to the survey study carried out through the same market survey company in 2020 [9], the prior study included people who endorsed at least 10 occasions of online gambling during the past year, whereas only one gambling occasion was theoretically sufficient for inclusion in the present study.

Web panel members of Ipsos are regularly invited to take part in market surveys and similar. For the completion of a survey such as the present one, the participant receives credits worth around one Euro, which can be transferred into goods within the web shop of Ipsos. Participants were fully anonymous and provided electronic informed consent before proceeding to the survey.

Given the similarity in design and topic with previous papers in the present setting, with items about self-exclusion worded in similar ways, the paper also presents below a comparison with three previous papers [9, 10, 12], regarding the rates of self-exclusion reported, the characteristics of the populations studied, factors associated with the reporting of self-exclusion, the rates of breaching (where applicable) and the types of gambling reported during self-exclusion (where applicable).

The study was approved by the Swedish Ethical Review Authority (File no. 2022-01332-1, approval date March 29, 2022).

### Study variables

The survey included the reporting of whether the respondent had ever gambled for the past 12 months, on each of the gambling types displayed in Table 1. The past-year reporting is in line with prior research used here for comparison [9, 10]. Gambling problems were assessed using the Problem Gambling Severity Inventory (PGSI, [13]), also used in similar previous studies [9, 10], as well as in regular Swedish health care surveys [14]. The PGSI comprises nine items measuring gambling behaviors and gambling-related problems, rating from ‘never’ (score 0) to ‘almost always’ (score 3), with a total score of 0–27. Total score representations are as follows: no-risk/no problem gambling (sum of 0), low-risk gambling (sum of 1–2), moderate-risk gambling (sum of 3–7), and problem gambling (sum of 8 or more), as used in previous studies [9, 10, 14]. Other items included gender, age (in age groups), living conditions (dichotomized in analyses as whether the respondent was living completely alone, without children, or not), main occupation (dichotomized into working/studying vs others), and whether the respondent had ever been prescribed treatment (therapy and/or medication) for poor mental health. The question about self-exclusion specifically referred to the *Spelpaus* service in use since 2019, and following questions asked about how long the chosen self-exclusion period was, whether the respondent gambled in any way during that self-exclusion, and, if yes, on which gambling types.

**Table 1:** Comparison of respondents reporting self-exclusion or no self-exclusion. Chi-square test

	Self-excluded (n = 135), n (%)	Never self-excluded (n = 1,359), n (%)	*p* value
Female gender	64 (48)	516 (38)	0.03
Age group (years)			
18–24	18 (13)	93 (7)	< 0.001
25–29	31 (23)	191 (14)	
30–39	38 (28)	276 (20)	
40–49	26 (19)	270 (20)	
50–59	12 (9)	267 (20)	
60–69	8 (6)	175 (13)	
70 +	1 (1)	87 (6)	
Ever prescribed mental health treatment	64 (48)	365 (27)	< 0.001
Living alone without children	27 (20)	352 (26)	0.13
Working/studying	122 (90)	1080 (79)	< 0.01
Gambling problems, PGSI*			< 0.001
None	17 (13)	813 (60)	
Low risk	10 (7)	262 (19)	
Moderate-risk	32 (24)	157 (12)	
Problem gambling	75 (56)	123 (9)	
Gambling problems, PGSI, moderate-risk or problem gambling**	107 (80)	285 (21)	< 0.001
Past-year gambling			
Online casino	110 (82)	478 (35)	< 0.001
Land-based casino	45 (34)	135 (10)	< 0.001
Online horse betting	74 (55)	665 (49)	0.19
Land-based horse betting	34 (25)	269 (20)	0.14
Sports live betting	68 (51)	632 (47)	0.35
Sports non-live betting	60 (45)	684 (50)	0.22
Sports (any)***	79 (59)	887 (65)	0.12
Online poker	44 (33)	253 (19)	< 0.001
Land-based poker	33 (24)	158 (12)	< 0.001
Land-based gambling machines	53 (40)	189 (14)	< 0.001
Online bingo	61 (45)	304 (22)	< 0.001
Gambling within video games	49 (36)	191 (14)	< 0.001

*Five participants with missing data for gambling problems

**Two individuals with missing data for gambling problems

***Participants reporting sports betting either as live betting or non-live betting, i.e. a variable collapsing the data from the prior two variables

### Statistical analyses

Participants reporting self-exclusion were compared to those who did not, using chi-square analyses, and likewise, among those reporting self-exclusion, participants reporting breaching were compared to those who did not. Variables which demonstrated significant associations with self-exclusion were entered in a non-stepwise logistic regression analysis, with lifetime self-exclusion as the dependent variable, in order to find independent associations with self-exclusion. Within the smaller group of those reporting self-exclusion, no multivariate regression was carried out with respect to breaching, as the absolute number of participants and thereby statistical power were considered to be limited.

## Results

From a total of 1505 respondents reporting past-year gambling, the majority were men (*n* = 914, 61 percent), 588 were women (39 percent), and three individuals reported ‘other/non-binary’ gender.

A total of 135 respondents (nine percent) reported experience of voluntarily self-excluding through the *Spelpaus* service. Response to the self-exclusion item was missing for eleven individuals. Among self-excluders, 21 percent (*n* = 28) reported a one-month self-exclusion, 25 percent (*n* = 33) reported three months, 24 percent (*n* = 32) reported six months, and 24 percent (*n* = 33) reported a 12-month self-exclusion, whereas six percent (*n* = 8) preferred not to answer.

Differences between self-excluders and the remaining gamblers are reported in Table 1. In logistic regression (Table 2), gambling problems and past-year online casino gambling remained significantly associated with self-exclusion, while online poker gambling was negatively associated with self-exclusion.

**Table 2:** Characteristics of individuals reporting self-exclusion. Logistic regression

	Odds ratio	95% CI
Age	0.90	0.77–1.05
Female gender	0.91	0.59–1.40
Ever prescribed mental health treatment	1.46	0.95–2.26
Working/studying	1.63	0.82–3.27
Moderate-risk/problem gambling	8.17	5.00–13.34*
Online casino	3.79	2.25–6.38*
Land-based casino	1.53	0.87–2.69
Online poker	0.44	0.25–0.77*
Land-based machine gambling	1.50	0.91–2.46
Online bingo	1.11	0.69–1.77
Land-based poker	0.74	0.40–1.39
Gambling within video games	1.04	0.62–1.75

*p < 0.05

Among self-excluders, 65 (49 percent) reported having gambled during self-exclusion (whereas response on this item was missing for two individuals). The most common gambling types reported during self-exclusion were online casino (*n* = 40, 82 percent), sports betting (*n* = 23, 47 percent), lotteries (n = 21, 43 percent), restaurant casinos (*n* = 12, 24 percent), private gambling occasions (*n* = 4, 8 percent), illegal land-based gambling venues (*n* = 2, 4 percent), or other (*n* = 3, 6 percent).

Respondents who breached self-exclusion, compared to their counterparts, were significantly more likely to report moderate-risk or problem gambling (89 vs. 69 percent, *p* < 0.01), land-based casino (45 vs 24 percent, *p* = 0.01), online poker (47 vs. 19 percent, *p* < 0.001), land-based poker (36 vs. 13 percent, *p* < 0.01), online bingo (55 vs. 34 percent, *p* = 0.01), gambling within video games (52 vs. 21 percent, *p* < 0.001), and mental health problems (*p* < 0.01). No significant associations were observed with age (p = 0.43, linear-by-linear), female gender (52 vs. 43 percent, *p* = 0.30), living alone without children (12 vs. 25 percent, *p* = 0.06), working/studying (92 vs. 90 percent, *p* = 0.56), online casino (85 vs. 79 percent, *p* = 0.44), online horse betting (63 vs. 47 percent, *p* = 0.06), land-based horse betting (32 vs. 19 percent, *p* = 0.08), land-based gambling machines (47 vs. 31 percent, *p* = 0.06), and any sports betting (66 vs. 51 percent, *p* = 0.09).

A comparison with previous *Spelpaus* data, and a comparison of data describing the breaching of self-exclusion in relation to previous studies, is presented in Table 3. For example, study designs, rates of self-exclusion, rates of breaching one’s self-exclusion and gambling types used when gambling during self-exclusion, are data which are compared to previous comparable studies.

**Table 3:** Comparison of four web panel surveys addressing *Spelpaus* self-exclusion in Sweden 2019–2022

Study	Håkansson and Henzel [10]	Håkansson and Widinghoff [9]	Claesdotter-Knutsson and Håkansson [12]	Present study
Design	Online web survey	Online web survey	Online web survey	Online web survey
Time period	Sept, 2019	May, 2020	March, 2021	May, 2022
Population	Web panel members with or without gambling (*n* = 2002)	Web panel members, online gambling 10 + times past year (*n* = 1007)	Web panel members, sub-group of 'gamblers’* (*n* = 1064)	Web panel members, 1 + occasion of gambling past year (*n* = 1505)
Rates of *Spelpaus* self-exclusion (ever, since start of system in 2019)	4%	7%	5%	9%
Correlates of *Spelpaus* self-exclusion	Younger, problem gambling	Moderate-risk/problem gambling, past-year online casino, absence of past-year sports betting	–	Moderate-risk/problem gambling, past-year online casino, absence of past-year online poker
Rates of breaching of *Spelpaus* self-exclusion	–	38%	–	49%
Most common gambling types reported during self-exclusion	–	52% online casino, 36% land-based lotteries, 16% sports betting, 21% online lotteries, 20% ‘other’	–	82% online casino, 47% sports betting, 43% lotteries 24% restaurant casinos

*All participants not endorsing the alternative 'do not gamble, neither before nor during COVID-19’

## Discussion

The present study, carried out when a new nationwide self-exclusion service had already been in use for almost 3.5 years, demonstrated that nine percent of past-year gamblers had ever used this self-exclusion service. Although head-to-head comparisons with other studies are difficult, a comparison with three previous online surveys from the present setting, addressing the same self-exclusion service, indicates that its popularity may have increased. On the other hand, breaching of one’s self-exclusion appears to be common, and may have increased to some extent since a comparable survey one year earlier. Thus, the present study displays the high level of use of this type of harm reduction tool in gamblers, but also presents challenges that need to be addressed in future policy work in this area.

In the study carried out in March 2021, primarily addressing potential COVID-19-related changes in the gambling market, five percent of gamblers had ever self-excluded through the Swedish self-exclusion service [12]. In the study from 2020, seven percent of participants with 10 occasions or more of past-year online gambling had ever self-excluded [9]. In the present study, around nine percent endorsed a history of self-exclusion. Given the growing experience of self-exclusion since the introduction of this service in 2019, it may seem unsurprising that the proportion having used the service was higher in the present study. Although the figures cannot be directly compared head to head, due to some differences in the samples assessed, it can be hypothesized that the present self-exclusion service is increasingly known by the general public over time. Likewise, however, the proportion of self-exclusion breachers was somewhat higher in the present study; 49 percent compared to 38 percent in the 2020 survey [9].

The breaching of self-exclusion is not a surprising finding, and constitutes a well-known limitation to self-exclusion services overall [7]. The specific interest in the present findings, however, is the fact that the Swedish self-exclusion service has been designed specifically to provide exclusion from a very broad range of gambling operators, including all companies with a license to operate in the Swedish jurisdiction, and involving gambling types as diverse as land-based casino gambling, online casino or card gambling, or sports betting conducted online or in bookmaker venues. It is also of great relevance to study self-exclusion and the breaching of self-exclusion in such a highly online-based gambling setting, as the online modality may present specific challenges (but also potential advantages) in comparison to more traditional self-exclusion preventing an individual from entering a land-based gambling venue. It has been discussed that the shame and stigma of actually entering a gambling setting, from which one has made a public commitment to avoid, would be a strong factor preventing an individual from gambling relapse [15]. This hindering factor may not be in play to the same extent in an online setting, where possibly both the gambling problem, the attempt to self-exclude from it and the attempts to relapse despite it, may happen in full secrecy through one’s personal smartphone or similar. While the online gambling setting theoretically may also widen the possibilities to self-exclude, enabling an authentication and log-in procedure and the technical possibilities to exclude a person from entering, the ease of finding new gambling opportunities in secret may present a limitation to the type of highly online-based self-exclusion services considered here.

The present study again demonstrates the particular challenges of online gambling, over and above the well-known risks of gambling overall. Online casino and online live-based sports betting constitute a more accessible and more rapid gambling modality than traditional venue-based land-based gambling [16]. In addition to that, as demonstrated by the present and previous study gambling [9], online gambling appears to be particularly difficult to self-exclude from, due to the availability of online-based operators outside the jurisdiction of *Spelpaus*. The clinical significance of this is obvious and has been confirmed in recent reports from a treatment unit [11]. Thus, while patients with a gambling disorder appear to self-exclude to a relatively large extent, it is obvious from such clinical data that their need for treatment remains, based on the risk of breaching, pointing to the further need for accessible treatment despite the existence of self-exclusion services. Given the limitations of self-exclusion it could be argued that a self-exclusion service may need to provide further support above the actual self-exclusion procedure. For example, a self-exclusion may need to add a motivational intervention or an advice for treatment, or guidance about where to seek formal treatment. Thus, one implication from the present findings might be a need for more extensive efforts than only the self-exclusion as such.

In addition, online casino was the only specific gambling type which was associated with the reporting of having self-excluded. Thus, although a number of gambling types with addictive potential were included in the study, online casino appears to have a specific position as gambling behavior surrounding the decision to self-exclude. Interestingly, for example, sports and horse race betting were not more commonly reported by responders reporting self-exclusion, than among others. Also, while other gambling types were more common in self-excluders, they were no longer associated with self-exclusion when controlling for online casino and variables describing gambling problems. Again, this points to the importance to continue to follow the addictive properties of online casino, also given its predominance in treatment-seeking clients in this setting [11].

Also, mental health problems were more common in self-excluders than in other respondents. This is in line with previous data [9], but in both studies, mental health problems were no longer associated with self-exclusion after controlling for gambling-related and other variables. In this study, mental health problems were measured with a single question about therapy history, as opposed to a previously used six-item scale [9]. Nonetheless, it appears that mental health problems may have a weaker link to self-exclusion than the actual gambling-related behaviors themselves.

The gambling types used during self-exclusion are comparable to those reported in 2020, although the absolute number of participants reporting breaching at that time was low [9], and therefore exact proportions of gambling types should be interpreted with caution. However, in the present study, 82 percent of those reporting breaching of self-exclusion reported online casino, compared to 52 percent in the previous study. Sports betting was also somewhat more common, 47 percent in the present study compared to 16 percent in the previous study. Likewise, restaurant casinos were reported by 24 percent in the present study, and although this absolute number is low, it was lower in the previous study [9]. It may be difficult to conclude whether these proportions represent any actual changes in the gambling market accessed for self-excluded individuals who relapse into gambling, and here, further studies in larger datasets may be needed. However, an overall concentration of problem gambling to online casino primarily, and also to sports betting, has been seen among treatment-seeking patients [11], and the high proportions of online casino and sports betting in self-exclusion breaching could be seen as consistent with this.

The factors associated with having breached self-exclusion were also reported as groupwise comparisons, but due to the relatively small sample size in that sub-study, a more extensive statistical analysis of these factors could not be carried out. However, the groups of self-excluders reported breaching and those who did not, differed with respect to gambling-related variables; gambling problems were even more common in those reporting breaching than in other self-excluders, and several gambling types were more commonly reported by those reporting breaching, and even more gambling types had a marginally significant difference between the groups. Overall, this points to a more intense gambling patterns in the respondents who reported to have gambled during their self-exclusion than among those who remained gambling-abstinent during self-exclusion. It further emphasizes the need to study the risk factors of breaching one’s self-exclusion and how policy makers can improve this harm reduction tool in order to increase its efficacy in individuals with intense gambling practices. Here, qualitative research may also provide important information about the psychological processes behind self-excluding and the factors associated with the decision to breach one’s self-exclusion.

Voluntary self-exclusion through the present nationwide, multi-operator model appears to be a popular harm reduction tool in Sweden; even though the sample addressed in the present study was not necessarily gambling as frequently as in the study from 2020 (past-year gambling in the present study compared to past-year gambling on 10 or more occasions in the 2020 study), the proportion reporting self-exclusion was not lower. It remains to be understood what reasons are behind self-exclusion in individuals who do not fulfill the criteria of gambling problems. Previous research describing the reasons for self-excluding—although from a specific gambling operator—indicates that reasons include a range of symptoms or consequences typically associated with a GD or a problematic gambling behaviour, but a very common reason reported is the willingness to prevent one’s own gambling [6]. Thus, it remains to be studied further how such preventive measures apply both to individuals with an established or earlier gambling problem, or hypothetically also to other people. It also cannot be excluded that self-exclusion may attract family members or partners of individuals struggling with a GD, in order to provide motivational or moral support to the patient, or potentially in order to prevent gambling on other family members’ identity. While this goes beyond the scope of the present study, it merits further research to examine why people without measurable gambling problems may choose to self-exclude. In addition, the present self-exclusion system prohibits gambling operators to address a self-excluded individual with direct postal or electronic advertising. Potentially, this may also add to the rationale behind self-excluding in the absence of gambling problems. Overall, research needs in the area remain, and the rates of self-exclusion and breaching demonstrated here highlight the need for further research, including actual effect studies of self-exclusion regarding gambling and mental health after choosing to self-exclude.

The present study has some limitations, primarily due to its design as a web survey, where the format is required to be kept brief, and where more thorough diagnostic examination cannot take place. The subjective, self-report-based information about self-exclusion may also be limited by recall bias. Nonetheless, the items addressing self-exclusion have been worded in the same way as in previous studies [9, 10, 12], making comparisons possible.

## Conclusions

S elf-exclusion remains a well-established and common harm reduction tool applied by individuals with gambling problems, but breaching of one’s self-exclusion is common, possibly somewhat more common than in previous comparable research from the same setting [9]. Breaching of self-exclusion may be seen as a natural consequence of a GD, and something common among individuals with gambling problems and with intense gambling practices. This may also be particularly challenging in a strongly online-dominated gambling market. In Sweden, online gambling represents the vast majority of gambling patterns for which patients seek treatment [11]. This may lead to a larger risk of self-exclusion breaching than in a more traditional, land-based gambling market. Aiming towards lower risk of self-exclusion breaching, further policy work may be needed in order to improve regulations around self-exclusion, such that overseas gambling operators can more easily be kept outside the market.

## Data Availability

Data can be requested form the first author, and can be made available to researchers after a legitimate request.
